# MobileSkin: Classification of Skin Lesion Images Acquired Using Mobile Phone-Attached Hand-Held Dermoscopes

**DOI:** 10.3390/jcm11175102

**Published:** 2022-08-30

**Authors:** Abdurrahim Yilmaz, Gulsum Gencoglan, Rahmetullah Varol, Ali Anil Demircali, Meysam Keshavarz, Huseyin Uvet

**Affiliations:** 1 Mechatronics Engineering, Yildiz Technical University, 34349 Istanbul, Turkey; 2 Department of Business Administration, Bundeswehr University Munich, 85579 Munich, Germany; 3 Department of Dermatology, Liv Hospital Vadistanbul, Istinye University, 34396 Istanbul, Turkey; 4 Department of Metabolism, Digestion and Reproduction, The Hamlyn Centre, Imperial College London, Bessemer Building, London SW7 2AZ, UK; 5 Department of Electrical and Electronic Engineering, The Hamlyn Centre, Imperial College London, Bessemer Building, London SW7 2AZ, UK

**Keywords:** deep learning, hand-held dermoscope, lightweight architectures, mobile phone, skin cancer

## Abstract

Dermoscopy is the visual examination of the skin under a polarized or non-polarized light source. By using dermoscopic equipment, many lesion patterns that are invisible under visible light can be clearly distinguished. Thus, more accurate decisions can be made regarding the treatment of skin lesions. The use of images collected from a dermoscope has both increased the performance of human examiners and allowed the development of deep learning models. The availability of large-scale dermoscopic datasets has allowed the development of deep learning models that can classify skin lesions with high accuracy. However, most dermoscopic datasets contain images that were collected from digital dermoscopic devices, as these devices are frequently used for clinical examination. However, dermatologists also often use non-digital hand-held (optomechanical) dermoscopes. This study presents a dataset consisting of dermoscopic images taken using a mobile phone-attached hand-held dermoscope. Four deep learning models based on the MobileNetV1, MobileNetV2, NASNetMobile, and Xception architectures have been developed to classify eight different lesion types using this dataset. The number of images in the dataset was increased with different data augmentation methods. The models were initialized with weights that were pre-trained on the ImageNet dataset, and then they were further fine-tuned using the presented dataset. The most successful models on the unseen test data, MobileNetV2 and Xception, had performances of 89.18% and 89.64%. The results were evaluated with the 5-fold cross-validation method and compared. Our method allows for automated examination of dermoscopic images taken with mobile phone-attached hand-held dermoscopes.

## 1. Introduction

Skin cancer is one of the most frequent and dangerous diseases today and is often caused by ultraviolet (UV) rays [1]. In the previous ten years, there has been a 44% increase in skin cancer cases in the United States [2]. The sunlight, along with its benefits for human skin, causes many diseases such as skin cancer in case of overexposure. The increased risk of developing skin cancer makes early detection of the disease critical [3]. Melanocytes, which are specific types of skin cells, begin to proliferate uncontrollably due to damage from UV rays, which results in the formation of malignant tumors known as melanoma. Melanoma is responsible for over 75% of skin cancer-related deaths [4]. However, with successful and early diagnosis in skin lesions, metastasis can be prevented. As a result, early detection of melanoma must be realized immediately to avoid metastasis [5].

For skin cancer diagnosis, suspicious lesions are first visually examined with the naked eye by dermatologists. In order to increase the diagnostic success, dermoscopes, also known as epiluminescence microscopes, integrated with a magnification lens and polarized or non-polarized light sources have been developed to make the superficial and deeper patterns of the lesions more visible and to provide a clearer and artifact-free imaging [6]. Digital dermoscopes (such as Molemax (Derma Medical Systems, Vienna, Austria)) also have an integrated digital camera and are usually connected to a computer. Since they are connected to a computer, they are stationary, bulky, and occupy valuable space in the clinic. Furthermore, due to their high-cost, many clinics around the world do not have access to a digital dermoscope and cannot benefit from many up-to-date technologies such as modern cameras. It has also been shown that the accuracy of visual examination of dermoscopic pictures increases linearly with dermatologist’s experience [7].

Non-digital, hand-held dermoscopes, on the other hand, do not need to be connected to a computer. They can be integrated to a mobile phone to utilize its digital camera. They are commonly preferred in the clinic because they are small, portable, inexpensive and compatible with almost every mobile phone. Thus, they allow the expert doctor to benefit from current camera technology. However, since they are not integrated with a camera, artifacts such as blurring due to vibration of the hand can be seen in dermoscopic images.

Lesions that cause skin cancer contain specific patterns and structures of their own. With dermoscopic examination, patterns and structures can be distinguished better, and lesion diagnosis can be made through images with higher performance. Accuracy of lesion diagnosis also depends on the experience of dermatologists, as it is based on visual information. Similarly, the performance of traditional machine learning studies to classify skin lesions is dependent on numerous criteria, such as the technique for feature extraction. In addition, the workload for each lesion grows as the number of characteristics increases. Therefore, its clinical application is not feasible. Numerous methods, such as ABCD principles [8] and seven-point checklists [9], have been developed previously to diagnose skin lesions in daily clinical practice. Diverse approaches have been developed to classify the handcrafted features [10]. In addition, it has been demonstrated that approaches developed by merging multiple methodologies improve performance for different aims [11,12]. However, handcrafted features do not reach deep learning performance and are ineffective in daily clinical practice because of their complexity [13,14,15]. Due to the success of deep learning on images, many segmentation and lesion classification studies have been performed for skin cancer using dermoscopic and clinical images. Since clinical images do not contain as much information about the lesion as dermoscopic images, clinical image-based diagnosis is less reliable due to decreased accuracy. Despite this shortcoming, the success of deep learning models on clinical images has reached the level of expert dermatologists [16]. Deep learning studies have focused more on dermoscopic images due to higher performance. One of the most significant steps in this regard is the International Skin Imaging Collaboration (ISIC) competition [17]. From 2016 to 2020, a total of 11 different challenges were held 5 times. According to the results of the ISIC competition, the most successful deep learning models produced an accuracy score well above the average expert dermatologist. In addition, studies were carried out to increase the success of the deep learning models by determining the region of interest automatically [18].

Another factor in skin cancer is skin type. Different parts of the world have different skin types. As the skin type changes, the characteristics such as the color structures and backgrounds of the lesions also change. Therefore, datasets such as HAM10000 or BCN20000 that contain different skin types were created [19,20]. Another contribution of the BCN20000 dataset is that it also includes artifactual images such as nails, mucosa, hypopigmented images, and lesions that do not fit into the image area. The HAM10000 and BCN20000 datasets provide more detailed information since the images are labeled with subtypes of lesions rather than only as malignant or benign. In clinical practice, the critical task is to classify the lesion as either benign or malignant since this is the deciding factor that determines whether to perform a biopsy or not. In order to provide this decision support mechanism, a patient-oriented, only binary-labeled dataset for melanoma diagnosis was also created [17].

Dermoscopic image datasets usually include metadata such as age, gender, and lesion area for each image. Multiple model studies were also carried out by including these metadata. Along with dermoscopic images, models in which clinical images were used together have also been developed. These studies have shown that accuracy increases with the use of additional data (e.g., metadata, clinical images) [21].

Recently, deep learning systems that will work as a decision support mechanism for the diagnosis of skin diseases have begun to be developed for people such as inexperienced dermatologists, nurses, and primary care doctors who are not experts in the field of dermatology [22,23]. Teledermoscopy was studied based on feature extraction with a small binary dataset [24]. In addition, feasibility of online teledermoscopy through a mobile application was also studied [25]. Similarly, they also provide preliminary information about the disease using a mobile phone at home. According to these studies, the performance in preliminary diagnosis can be increased with inexpensive and fast methods without the need for dermatologist examination. As can be seen, the number of studies on decision support mechanisms and prediagnosis on mobile and embedded devices has started to increase. There are many studies that use lightweight deep neural networks (DNN) for mobile devices, but dermoscopic images collected from digital dermoscopic datasets have mostly been used [26,27,28,29]. Due to the scarcity of doctors and the conversion of clinics such as dermatology to pandemic services, in the pandemic period, applications to guide the patient with mobile systems are being studied. There is a need for a cheap and practical decision support mechanism that can be used by non-dermatology specialists and can provide higher performance on skin lesions by using a camera integrated with hand-held dermoscope.

In this study, we collected a dermoscopic image dataset of skin lesions taken using a mobile phone-attached hand-held dermoscope. Each image was labeled as actinic keratosis (ak), seborrheic keratosis (sk), vascular lesion (vasc), dermatofibroma (df), basal cell carcinoma (bcc), squamous cell carcinoma (scc), nevus (nv), or melanoma (mel). Afterwards, a multi-class classification study was conducted on four DNN architectures. Each model was initialized with weights that were pre-trained on the ImageNet dataset. Then, transfer learning of the fully connected layer and fine-tuning of the convolutional layers were conducted. Performance metrics based on the 5-fold cross-validation results are presented for each model.

To summarize our main contributions: (1) For the first time, a dermoscopic dataset where each image was collected using a mobile phone attached hand-held dermoscope is presented. (2) The presented dataset was used to train four state-of-the-art deep learning models for the purpose of multi-class classification without the use of metadata and clinical images. (3) Performance metrics obtained from the trained models are presented and compared with each other in order to create a benchmark for the dataset.

## 2. Materials and Methods

**Figure 1:**
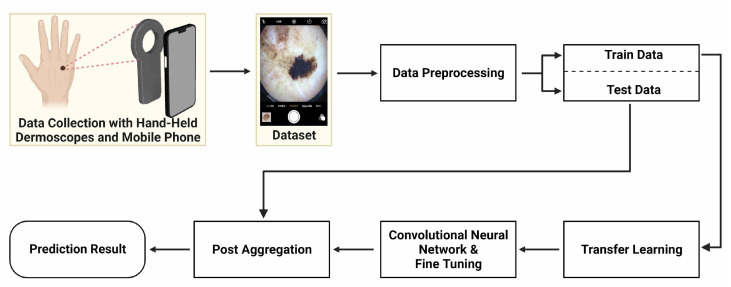
Main scheme of work, starting from data gathering to prediction results.

The presented method includes training with 5-fold cross validation and testing phases for the development of a deep learning model to classify skin lesions. Firstly, data preprocessing was applied to the dataset. For the training phase, four DNN architectures were initialized with weights that were pre-trained on the ImageNet dataset, and the fully connected layer was retrained using transfer learning [30]. Afterwards, fine-tuning was carried out by retraining the convolutional layers. Validation data were used in the training stage to obtain feedback about the performance of the model. In the testing phase, preprocessed test images were given to the deep learning models. A summary of the process is shown in Figure 1.

### 2.1. Mobile Dermoscopy Dataset

#### 2.1.1. Data Collection

**Figure 2:**
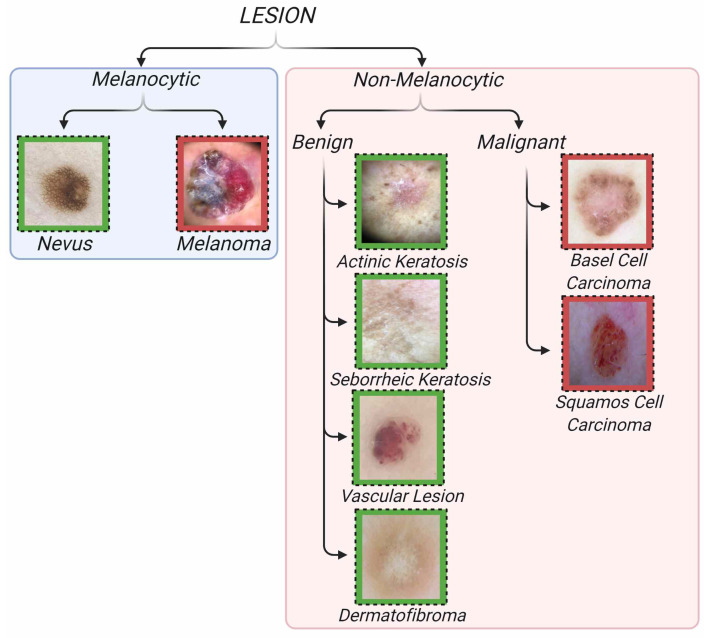
Classification of skin cancer lesions by groups and subgroups. Green background represents benign lesions, and red background represents malignant lesions.

Skin lesions can be divided into two classes: melanocytic and non-melanocytic. Both classes contain subtypes that are considered to be malignant lesions and classified as skin cancer. The proposed dataset includes the subtypes mel, the malignant form of melanocytic lesions and nv, the benign form of melanocytic lesions. Among the non-melanocytic lesions, six classes are included: ak, sk, df, and vasc lesions from the benign form and bcc and scc lesions from the malignant form of non-melanocytic lesions. The hierarchical structure of the dataset and example images of these classes are shown in Figure 2. Images were collected using a mobile phone-attached hand-held dermoscope from patients who came to the clinic with lesion complaints. All images were taken in JPEG image format with a 3gen Dermlite DL4 hand-held dermoscope and connection kit for iPhone 7. A standardized imaging process was followed using a similar illumination angle and intensity for each image. In order for the images to be artifact-free and in focus, the dermoscopic images were taken under appropriate and sufficient light. The manual focus feature was used to focus the camera and enough time was allowed for it to focus. The 1644 high-quality and artifact-free dermoscopic images were chosen from 1688 dermoscopic lesion images, which were collected from 2017 to 2021. Ethical approval of images is based on ethics review board protocols 21–82 (Istinye University, 1 November 2021). The dataset does not include metadata information such as gender or age. Image labeling was performed by reading the class of dermoscopic images collected from the patient reports. The dermoscopic images were collected by an expert dermatologist with 20+ years experience in dermatology. All melanoma cases are biopsy-proven. Most other lesion types are follow-up lesions. The dataset includes dermoscopic images and the type of lesion for each image. While the dataset was being created, a considerable attention was paid into keeping it balanced. A common problem for imbalanced datasets is that models tend to memorize classes with a large number of images and miss classes with a small number of images. In order to create a balanced dataset, images with excessive disturbances (e.g., blurry images) and artifacts that doctors have difficulty in diagnosing are not included in the dataset. However, images that do not affect the doctor’s diagnosis despite the presence of artifacts were included in the dataset. Furthermore, images of the same lesion taken from different angles and distances were also included. The lesion types, lesion names, class numbers, and training, testing and total sample sizes for each class of Mobile Dermoscopy Dataset are shown in Table 1.

#### 2.1.2. Data Augmentation

**Figure 3:**
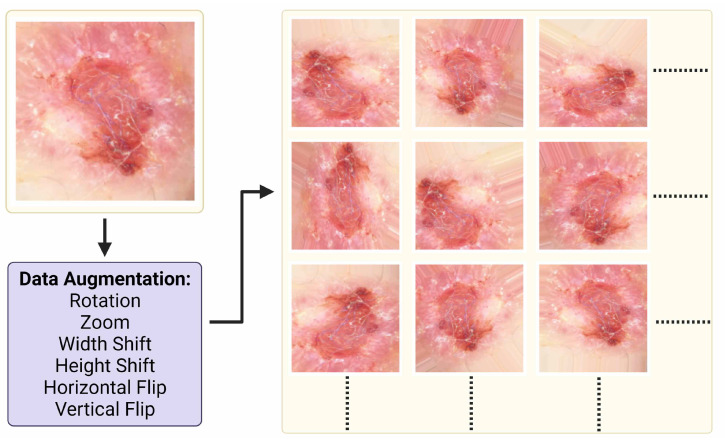
Data sample, data augmentation and output samples with respect to data augmentation settings.

Data augmentation techniques reduce overfitting and increase the performance of the models [31]. In order to increase the number of images in the dataset, the lesion area was cropped manually, and the images that contain the lesion were added to the dataset. In order to increase the training data, an image generator was created. The image generator for augmentation accepts a batch of images, applies augmentation techniques on each image, and replaces the original images with augmented images. Since the lesions are invariant when rotated and moved at any angle, six different augmentation methods were applied to the images: rotation, zoom, width and height shift, vertical and horizontal flip. It has been shown that these techniques increase the performance in image classification [32]. Images are augmented with a 45-degree rotation range, 0.2-percent focusing range, and 0.2-percent width and height shift. Example patch images of an augmented image from the dataset are shown in Figure 3. The parameter names and values of the data augmentation techniques are shown in Table 2.

### 2.2. Deep Learning Model

Following the high success rates of DNNs against traditional methods, they have been widely applied for classification of medical images as well. Particularly, convolutional neural networks (CNNs) can be trained to recognize complex patterns that are hard to model using hand-crafted features. In this study, a feature detection network was developed using CNNs, and then a fully connected layer that learns which feature is associated with which lesion was trained. The classification error was minimized by using the backpropagation algorithm using the training dataset [33]. Thus, the deep learning model was optimized, and the classification model with the highest performance was revealed.

#### 2.2.1. Deep Learning Architectures

There are many architectures that are based on different approaches in deep learning models. Usually, these architectures are designed to be efficient for specific tasks. Different deep learning architectures such as Xception [34], MobileNetV1 [35], MobileNetV2 [36], and NASNetMobile [37] have been developed to achieve high performance with a low number of network parameters. For this purpose, depthwise separable convolutions are a very effective method to reduce model size and improve accuracy [38]. Depthwise separable convolutions have two separate layers instead of a full convolutional operator. The depthwise convolution as the first layer applies a single convolutional filter per input channel, and the pointwise convolution as the second layer is a 1 × 1 convolution filter. MobileNetV1 and Xception are built on depthwise separable convolutions for efficient mobile models and scaling up depthwise separable filters. MobileNetV2 architecture, which is an improved version of the MobileNet architecture [35], differs in that it reduces the computational cost and is a smaller model. Depthwise separable convolution, shortcut connections, inverted residuals, and bottleneck layers are the innovations and structures that make MobileNetV2 more successful than its predecessor [36]. NASNetMobile uses the neural architecture search (NAS) with ScheduledDropPath technique and a mobile version of the NASNet model [39].

#### 2.2.2. Transfer Learning and Fine-Tuning

**Figure 4:**
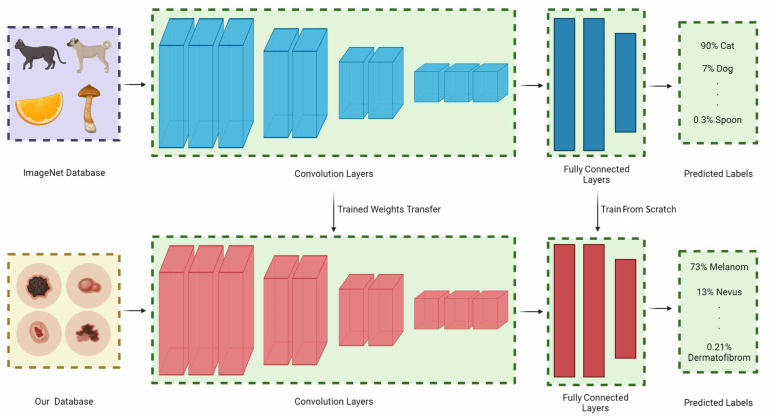
Overview of transfer learning process. The weights obtained on the ImageNet dataset are transferred to the convolution layers. The weights in the fully connected part are retrained. After optimization, the four deep learning models have two 128 node dense layers and one dropout layer with a 0.2 ratio as fully connected layers.

Transfer learning is a process in which particular layers are frozen and particular layers are retrained by implementing pre-trained networks on different large-scale datasets [40]. Depending on the dataset and the purpose of the pre-trained network, the retrained layers can be changed. The transfer learning concept is shown in Figure 4. The ImageNet dataset is the most commonly used dataset for obtaining the pre-trained weights [31]. The ImageNet dataset can be considered a universal feature resource because it contains 21841 synsets and more than 14 million images. The effectiveness of transfer learning using pre-trained models with ImageNet has been demonstrated in many applications. In this study, four deep learning architectures pre-trained with the ImageNet dataset was used.

#### 2.2.3. Network Implementation

In deep learning models, images are scaled to a lower resolution because high-resolution images take longer to process, and there exists a memory problem. Therefore, the collected images were scaled to 299 × 299 × 3 pixels for the Xception model and 224 × 224 × 3 pixels for MobileNetV1, MobileNetV2, and NASNetMobile. Since taking images by continuously accessing the memory during the training process causes a slowdown in model training, the RGB values of the images were converted to the array format of the NumPy library and saved. Then, training was done through these NumPy arrays [41]. In order to make the deep learning model suitable for our dataset, additional neural network layers were added to the end of the deep learning architectures. The GlobalAveragePooling layer was added to the end of the deep learning architectures, and then the Dropout layer was added with a ratio of 0.2 to implement architectures for this classification case. With the flatten layer, the image coming from the architecture is converted into a form to be processed in the fully connected layer. The fully connected layers of ImageNet were deleted. At the end of the deep learning model, a 128-node dense layer was added. Then, similarly, a 128-node dense layer and a dropout layer with a 0.2 ratio were added. Finally, the output layer with eight nodes was added. For detailed information about the four neural networks, see the Supplementary Materials. Since the classification problem includes eight classes and will be run on mobile platforms, the aim was to increase the performance with the least possible number of parameters. Pre-trained deep learning architectures on the ImageNet dataset were used with 2-stage training for fine-tuning the model. In the first stage, all the layers of the deep learning architecture were frozen. Only the fully connected block added to the end of the architecture was trained. Thus, it was ensured to select according to the universal features extracted from the ImageNet dataset. In the second stage, fine-tuning was carried out. At this stage, all model layers were included in the training, and the model that would reveal the highest performance was obtained by optimizing the universal features extracted from the ImageNet dataset. The dynamic learning rate approach was used to optimize the learning rate during the training using the ReduceLROnPlateau function. If the validation performance did not decrease every two epochs, the learning rate was reduced by 0.1 to accelerate learning. The Keras library was used over the Python programming language to train the models. Models were run on a system using Nvidia GTX1080Ti with 11GB memory, an AMD Ryzen Threadripper 1950X processor, and 32 GB RAM. A two-step 100 epoch model training was used in this study. The average training time of the deep learning model for 100 epochs of fine-tuning is about 2 h.

#### 2.2.4. Testing

There are different approaches to measure the success and robustness of the trained model, such as k-fold cross-validation and classification metrics. With k-fold cross-validation, the training dataset is divided into k parts, and k models are created. One fold is the validation dataset, and the rest is the training dataset. By mixing the training and validation data of the dataset in this way, the average model performance can be measured by eliminating the effect of randomness in the selection of training data. For the success criteria of the model, there are many metrics in classification problems. In this study, the accuracy (*Acc*), precision (*Prec*), and $F_{1}$ score of four models as a result of k-fold cross validation were calculated on test data. $F_{1}$ score was used to measure the balance of positive and negative prediction rates of the model. The formulas for these classification metrics are shown in Table 3.

## 3. Results

Table 4 shows the classification performance of the deep learning models. The skin lesion classification model based on the MobileNetV1, MobileNetV2, NASNetMobile, and Xception architectures, developed with eight outputs, were performed with 5-fold cross validation. The average Acc, Prec, and $F_{1}$ score values of the 5 models obtained with SD and 95% confidence intervals (CI) are 76.96% (±2.60, (74.7, 79.2)), 77.94% (±2.93, (75.4, 80.5)), and 77.45% (±2.76, (75, 79.9)) for MobileNetV1, respectively. The average Acc, Prec, and $F_{1}$ score values of the 5 models are 89.18% (±1.13, (88.2, 90.2)), 88.13% (±2.81, (85.7, 90.6)), and 87.38% (±2.52, (85.2, 89.6)) for MobileNetV2, respectively. The average Acc, Prec, and $F_{1}$ score values of the 5 models are 77.21% (±1.22, (76.1, 78.3)), 78.04% (±1.33, (76.9, 79.2)), and 77.62% (±1.24, (76.5, 78.7)) for NASNetMobile, respectively. The average Acc, Prec, and $F_{1}$ score values of the 5 models are 89.64% (±1.89, (88, 91.3)), 89.99% (±1.73, (88.5, 91.5)), and 89.81% (±1.8, (88.2, 91.4)) for Xception, respectively.

**Figure 5:**
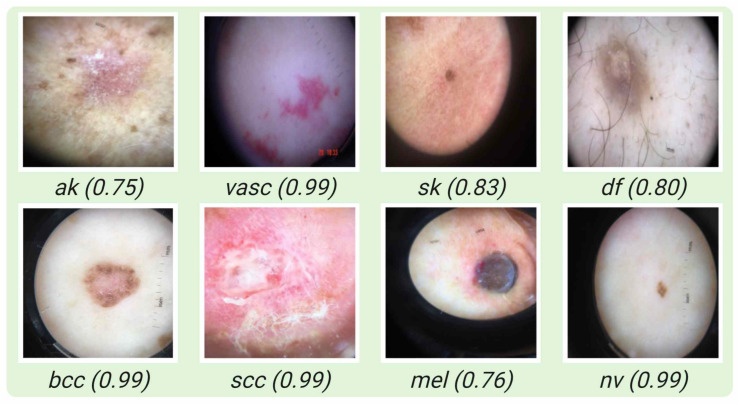
Samples of correctly classified images with their corresponding probability.

**Figure 6:**
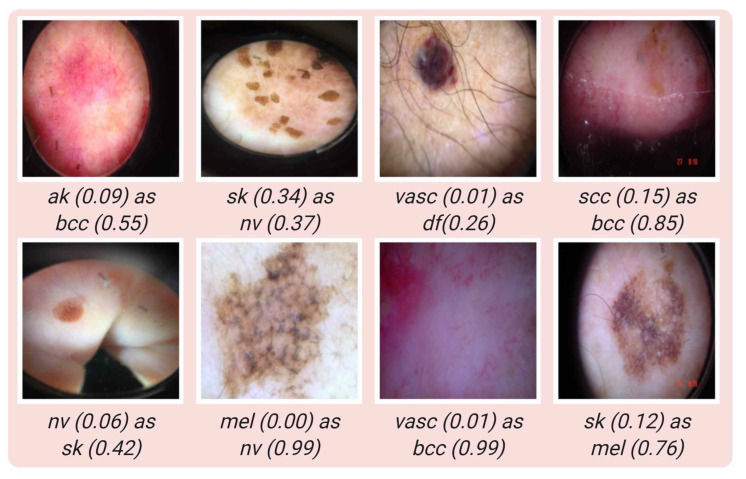
Samples of misclassified images, true classes with prediction values of true class and false predicted classes with corresponding false prediction values.

Some samples where the model classifies correctly for each class are shown in Figure 5. In Figure 6, some examples of incorrect classification are shown for each class. In Table 5, the performance of four deep learning models with their standard deviations (SD) for each class are given.

## 4. Discussion

Studies on skin cancer can be classified under three categories: studies with clinical photographs, studies with dermoscopic photographs, and studies with multiple models. In studies with clinical photographs, the images collected by directly taking the skin image are processed. Most of the images collected from the skin illuminated by a polarized or non-polarized light source are processed in studies with dermoscopic photographs. In multi-model studies, results are obtained by combining different models, including clinical, dermoscopic, and metadata information. Table 6 shows our dataset specifications and other open access skin lesion datasets.

There are many studies comparing dermatologists and developed deep learning models in macroscopic image analysis. In these studies, while deep learning models gave better results than beginner and intermediate-level experienced dermatologists, they did not produce better results in all comparisons when compared to expert dermatologists [49,50]. There are two major breakthroughs in this field. The first is Esteva et al.’s work [16], which includes 129,450 images containing 2032 classes. They developed a deep learning model based on the Inception v3 CNN architecture by reducing 2032 classes to 3 and 9 main classes according to a taxonomy tree and compared it with two board-certified dermatologists. They achieved a similar success rate to the dermatologists. The other work is by Han et al., where they developed the ResNet152 CNN architecture for a dataset containing 19,398 images of 12 different skin diseases [42]. As a result of the comparison made with 16 dermatologists, 10 of whom were professors and 6 were clinicians, the developed deep learning architecture gave much better results than dermatologists, especially in terms of accuracy. The use of clinical images aims to make decisions by using skin images directly without the use of any tools. In this process, skin lesion images taken with any mobile phone can be uploaded to a mobile phone application, and a prediction result can be produced. Since it is an inappropriate and expensive process for patients to purchase a digital dermoscope device or a hand-held dermoscope, datasets and studies created in this field stand out in producing results using a mobile phone in home conditions. However, low performance and model reliability are the biggest problems. In addition, the model estimation is not stable because the images are affected by parameters such as the light angle, intensity, skin type, and patterns that are not visible enough.

Most of the studies on skin cancer have been carried out using dermoscopic images [51]. Their performance is much higher than macroscopic images since superficial and deeper patterns can be selected in dermoscopic images. The majority of studies in the field of dermoscopic images are carried out on datasets shared under ISIC. The first challenge was carried out in 2016 on 1279 dermoscopic images [44]. In the challenge in which 25 teams participated, dermatologists obtained similar results with the best model, and thanks to the fusion algorithm developed, a specificity value of 76% was obtained against the specificity value of 59% of dermatologists [45]. In addition, an area under of the curve (AUC) value of 0.86 was obtained from the fusion algorithm, while the AUC value of the dermatologists remained at only 0.71. In the challenge in 2017, three different tasks were performed, in which lesion segmentation, dermoscopic feature classification, and three different lesion classification were performed [46]. In 2018, similar tasks were performed on 12,500 images as in 2017. For the challenge held in 2018, the performance of 511 participants from 63 different countries was measured [47]. While the top three deep learning models had an average sensitivity of 86.2%, the best model had a sensitivity value of 88.5%. Despite the deep learning models, the average participant sensitivity remained at only 79.2%, while the expert dermatologist sensitivity was 81.2% [48]. Brinker et al. also developed a deep learning model trained with dermoscopic images and classified clinical images with this model. Comparing the results of the deep learning model with the performance of 145 dermatologists, they showed that the deep learning model performed better than doctors in clinical images, even though it was trained on dermoscopic images [50]. As seen from the most extensive datasets and participants, skin lesion diagnosis is highly related to the experience of examiners. Therefore, even if dermoscopic images are used, the use of decision support mechanisms will be effective in increasing the success of both experienced and inexperienced dermatologists. However, great strides can be taken in the success of diagnosis with an inexpensive method based on mobile or embedded devices that can be accessed by many medical institutions, including primary health care institutions.

In multi-model studies, datasets containing dermoscopic images, clinical images, and metadata were used in different skin cancer studies using different combinations. The most comprehensive study in this area is the study by Yap et al., in which they tried six combinations of macroscopic, macroscopic + metadata, dermoscopic, dermoscopic + metadata, dermoscopic + macroscopic, and dermoscopic + macroscopic + metadata [21]. This study showed that the model trained with only dermoscopic images gave outstanding results compared to macroscopic and macroscopic + metadata models, which did not include dermoscopy images. Each additional dataset added on top of the dermoscopic dataset contributed to the performance and stability of the model. In a study by Pacheco et al., two models were trained using images collected by mobile phones. The first is the scenario where only clinical images are used, and the second is the scenario where clinical images and clinical features are used as multiple models. As seen from this study, the scenario using only clinical images produced similar results to other publications that use clinical images. However, in the scenario where clinical images and clinical information were used, approximately 7% more success was achieved [52]. In another study, they developed a combined CNN-based model using clinical and dermoscopic images presented by Tschandl et al. In this study, when clinical and dermoscopic images were used together, the deep learning model produced better results than novice (<3 years) and moderately experienced dermatologists (3–10 years). However, it could not reach the success of expert dermatologists (>10 years) [53]. As seen from multi-model studies, inclusion of clinical images, dermoscopic images, or metadata increases accuracy. However, extra processing and data entry is required for each added dataset. That data entry is not possible in countries where the number of patients per doctor is high. In addition, in the case of using different datasets together, even if the model performances increase, results and predictions are produced at the extreme points. In this regard, the most stable prediction distribution is the studies in which only dermoscopic datasets are used, although the performance is slightly lower than the others.

As can be seen from the results, although dermoscopic images taken using a mobile phone integrated into hand-held dermoscopes contain large distortions such as blurring, a model with high performance can be obtained due to their high resolution. Recently, the most significant breakthrough with the work on mobile phones has come from the Google company. They developed a mobile dermatology application for mobile phones on skin lesions. According to the study, which was published as a landmark study of mobile application, a model developed using clinical images and metadata datasets with the secondary purpose for a total of 419 skin diseases and high performance for 27 common skin diseases was presented from 16,114 images [54]. Similarly, in a study involving 20 primary care physicians and 20 nurse practitioners, a total of 40 board-certified clinicians, by using artificial intelligence as a decision support mechanism, the success of clinicians increased from 48% to 58% for primary care physicians, and the success of nurse practitioners increased from 46% to 58% [22]. As can be seen from the studies, mobile phones will be actively used as a decision support mechanism in the following years. Thus, the performance of not only expert dermatologists but also other clinicians will be increased cheaply and practically. Our study presents a deep learning model for skin lesions using the four deep learning architectures, which can produce high results with low parameters for a mobile application by using dermoscopic images, which are datasets that produce successful results. The nv lesion is the most successfully classified lesion type. The majority of misclassified nv lesions were predicted as sk lesion. The df and sk lesion types also have the lowest success because of the small sample size and similarity with nv lesions, respectively. In addition, all misclassified scc lesions were predicted as bcc lesions, and the majority of misclassified bcc lesions were classified as scc lesions. The misclassification of lesions that are in the same group as malign or benign is not as bad as other error types. In addition, ak and vasc lesions can be classified with high accuracy. The most important lesion type, melanoma, has a high class accuracy around 90%. Finally, the decision support systems can be very effective in daily clinical practice with successful predictions. Both expert dermatologists and other clinicians will be able to use artificial intelligence as a decision support mechanism by using their mobile phones only with the help of hand-held dermoscopes.

## 5. Limitations

This study includes some limitations. First, the presented dataset was collected only from the western Turkish region. Therefore, Fitzpatrick’s skin types mainly include type 2 and type 3 skin types. Secondly, lesions collected from nails and mucosal regions were not included in the study. Third, the dataset does not include metadata and macroscopic images. A more stable and reliable model can be put forward by participating in the study of these datasets and increasing the number of data.

## 6. Conclusions

In the present study, a dataset containing dermoscopic images of eight skin lesions collected using a mobile phone-attached hand-held dermoscope has been presented to the literature. Deep learning models based on four deep learning architectures, which aim to produce high performance with few parameters for mobile phones, has been developed using this dataset. This deep learning model has the ability to be used over mobile phones as a decision support mechanism for both expert dermatologists and clinicians. In future studies, the dataset will be expanded and tested with other dermatologists to compare their performance with the deep learning model. In addition, examinations will be conducted on how much the performance of clinicians can be increased by using only hand-held dermoscope in decision support.

## Figures and Tables

**Table 1:** The lesion types, names, class number and dataset size.

Type	Lesion Name	Class Number	Training-Testing-Total Class Size
Non-Melanocytic Benign	Actinic Keratosis (ak)	1	38-10-48
Non-Melanocytic Benign	Vascular Lesion (vasc)	2	160-40-200
Non-Melanocytic Benign	Seborrheic Keratosis (sk)	3	143-36-179
Non-Melanocytic Benign	Dermatofibroma (df)	4	29-7-36
Non-Melanocytic Malignant	Basel Cell Carcinoma (bcc)	5	188-47-235
Non-Melanocytic Malignant	Squamous Cell Carcinoma (scc)	6	141-35-176
Melanocytic Malignant	Melanoma (mel)	7	124-31-155
Melanocytic Benign	Nevus (nv)	8	492-123-615
Total	-	-	1315-329-1644

**Table 2:** Data augmentation arguments and their range and values.

Settings	Values
Rotation Range	45
Zoom Range	0.2
Width Shift Range	0.2
Height Shift Range	0.2
Horizontal Flip	True
Vertical Flip	True

**Table 3:** Metrics and formulas used to measure model performance.

Metric	Formula
Accuracy	$\frac{TP+TN}{TP+FP+TN+FN}$
Precision	$\frac{TP}{TP+FP}$
$F_{1}$ Score	$2*\frac{\mathrm{Precision}\times \mathrm{Recall}}{\mathrm{Precision}+\mathrm{Recall}}$

**Table 4:** Mean values and SDs for weighted metrics of four deep learning models evaluated with 5-fold cross validation.

Metric	MobileNetV1	MobileNetV2	NASNetMobile	Xception
Accuracy	76.96%±2.60	89.18%±1.13	77.21%±1.22	89.64%±1.89
Precision	77.94±2.93	88.13%±2.81	78.04%±1.33	89.99%±1.73
$F_{1}$ Score	77.45%±2.76	87.38%±2.52	77.62%±1.24	89.81%±1.80

**Table 5:** Shows the class accuracies for each of the eight classes, along with their mean percentile performance and SD.

Lesion	MobileNetV1	MobileNetV2	NASNetMobile	Xception
ak	68.00% (±13.04)	80.00% (±0.00)	72.00% (±16.43)	66.00% (±20.74)
vasc	80.50% (±4.47)	90.50% (±2.82)	78.50% (±6.52)	91.00% (±4.18)
sk	52.78% (±7.61)	67.78% (±8.97)	56.11% (±6.63)	72.78% (±10.65)
df	37.14% (±12.78)	68.57% (±3.67)	40.00% (±25.56)	71.43% (±14.29)
bcc	65.11% (±8.85)	73.62% (±10.94)	61.70% (±7.82)	73.19% (±6.13)
scc	65.14% (±2.39)	89.71% (±5.75)	65.14% (±3.73)	85.71% (±4.95)
mel	85.81% (±7.77)	89.03% (±4.31)	85.81% (±7.43)	87.74% (±2.70)
nv	91.38% (±0.93)	91.87% (±2.64)	92.52% (±2.47)	91.00% (±4.18)

**Table 6:** Specifications of the largest open access skin lesion datasets in the literature and related studies.

Dataset	Study	Type	Comparison with Dermatologists	Dataset Size	Class Size	Dermatologists Number
Hybrid 1 *	[16]	Clinic	Yes	129,450	9	2
Hybrid 2 **	[42]	Clinic	Yes	19,398	12	16
$PH^{2}$	[43]	Dermoscopic	No	200	3	-
ISIC 2016	[44,45]	Dermoscopic	Yes	1279	3	8
ISIC 2017	[46]	Dermoscopic	No	2750	3	-
ISIC 2018	[19,47,48]	Dermoscopic	Yes	10,015	7	511
ISIC 2019	[19,20]	Dermoscopic	No	25,331	8	-
ISIC 2020	[17]	Dermoscopic	No	33,126	2	-
Mobile Dermoscopy	Own	Dermoscopic	No	1644	8	-

* ISIC Dermoscopic Archive, the Edinburgh Dermofit Library and Stanford Hospital. ** Asan, MED-NODE dataset and atlas site images.

## Data Availability

The data that support the findings of this study are available in the website at the link https://www.asillab.com/en/mlgroup, accessed on 15 July 2022.

## Supplementary Materials

The following supporting information can be downloaded at: https://www.mdpi.com/article/10.3390/jcm11175102/s1, Figure S1: The layers of fully connected block of deep learning models.; Figure S2: The layers of fully connected block of deep learning models with parameters.; Figure S3: The layers of convolution block of MobileNetV1 architecture.; Figure S4: The layers of convolution block of MobileNetV1 architecture with parameters.; Figure S5: The layers of convolution block of MobileNetV2 architecture.; Figure S6: The layers of convolution block of MobileNetV2 architecture with parameters.; Figure S7: The layers of convolution block of NASNetMobile architecture.; Figure S8: The layers of convolution block of NASNetMobile architecture with parameters.; Figure S9: The layers of convolution block of Xception architecture.; Figure S10: The layers of convolution block of Xception architecture with parameters. Reference [55] is cited in Supplementary Materials.
