# Supplementary material for: MobileSkin: Classification of Skin Lesion Images Acquired Using Mobile Phone-Attached Hand-Held Dermoscopes
Source: J Clin Med. 2022 Aug 30;11(17):5102. doi: 10.3390/jcm11175102 (PMC9457478; doi:10.3390/jcm11175102)
Supplement: Supplementary file 1 [file jcm-11-05102-s001.zip › Supplementary Figures.pdf]

All figures were visualized by using Netron visualizer [1].

[1] Roder, L. Netron, Visualizer for neural network, deep learning, and machine learning models. 2020. Available online: https://doi.org/10.5281/zenodo.5854962 (accessed on 5 June 2022). doi:10.5281/zenodo.5854962

Figure S1

The layers of fully connected block of deep learning models.

Figure S2

The layers of fully connected block of deep learning models with parameters.

Figure S3

The layers of convolution block of MobileNetv1 architecture.

Figure S4

The layers of convolution block of MobileNetv1 architecture with parameters.

Figure S5

The layers of convolution block of MobileNetv2 architecture.

Figure S6

The layers of convolution block of MobileNetv2 architecture with parameters.

Figure S7

The layers of convolution block of NASNetMobile architecture.

Figure S8

The layers of convolution block of NASNetMobile architecture with parameters.

Figure S9

The layers of convolution block of Xception architecture.

Figure S10

The layers of convolution block of Xception architecture with parameters.
