# Supplementary figures and images for: MobileSkin: Classification of Skin Lesion Images Acquired Using Mobile Phone-Attached Hand-Held Dermoscopes
Source: J Clin Med. 2022 Aug 30;11(17):5102. doi: 10.3390/jcm11175102 (PMC9457478; doi:10.3390/jcm11175102)

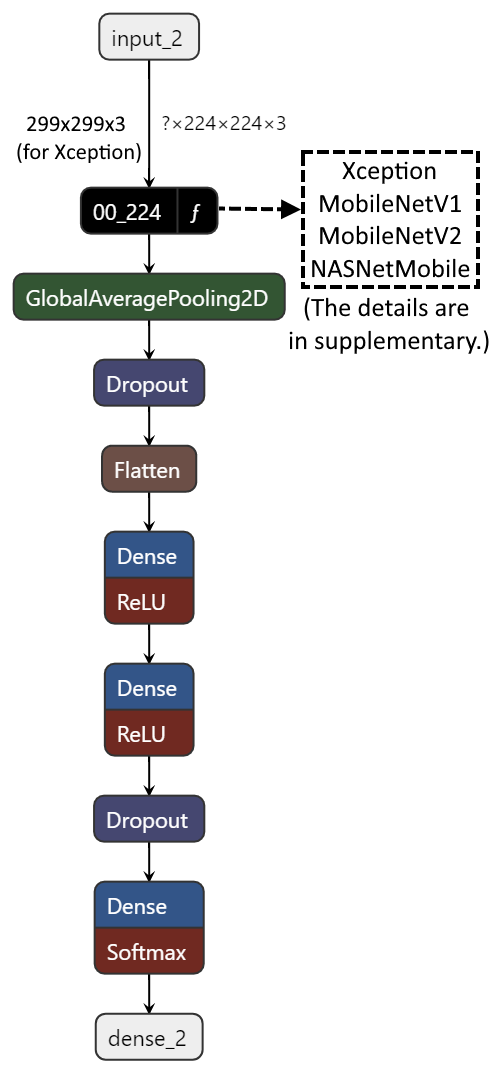

Supplement: Supplementary file 1 [file jcm-11-05102-s001.zip › Figure S1.png]

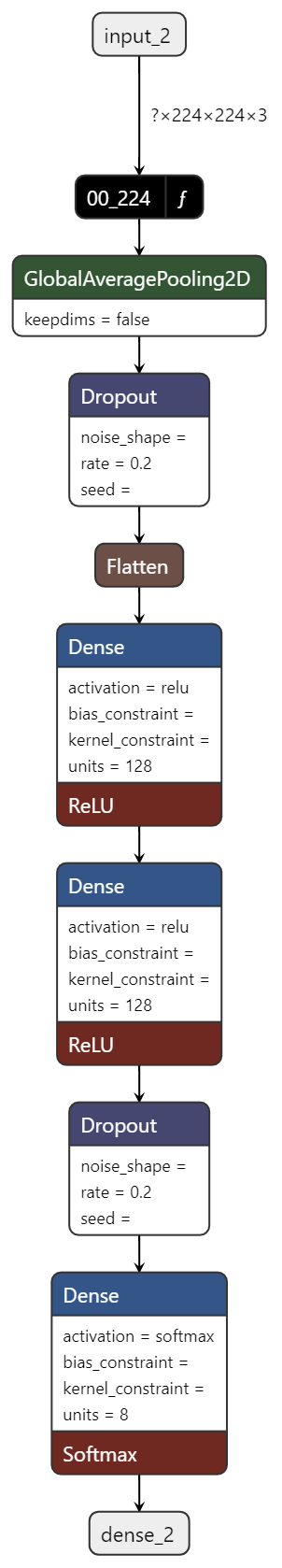

Supplement: Supplementary file 1 [file jcm-11-05102-s001.zip › Figure S2.png]

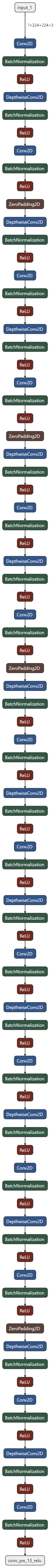

Supplement: Supplementary file 1 [file jcm-11-05102-s001.zip › Figure S3.png]

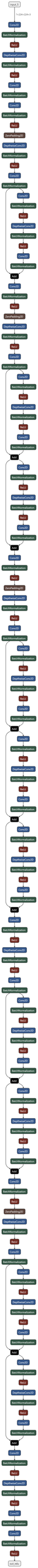

Supplement: Supplementary file 1 [file jcm-11-05102-s001.zip › Figure S5.png]

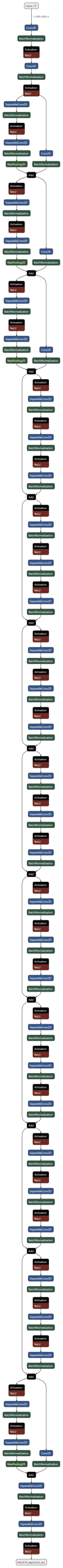

Supplement: Supplementary file 1 [file jcm-11-05102-s001.zip › Figure S9.png]
